# Recent Advances in Synthesis and Applications of MFe_2_O_4_ (M = Co, Cu, Mn, Ni, Zn) Nanoparticles

**DOI:** 10.3390/nano11061560

**Published:** 2021-06-13

**Authors:** Thomas Dippong, Erika Andrea Levei, Oana Cadar

**Affiliations:** 1 Faculty of Science, Technical University of Cluj-Napoca, 430122 Baia Mare, Romania; thomas.dippong@cunbm.utcluj.ro; 2 National Institute for Research and Development for Optoelectronics INOE 2000, Research Institute for Analytical Instrumentation Subsidiary, 400293 Cluj-Napoca, Romania; erika.levei@icia.ro

**Keywords:** transition metal, ferrites, magnetic nanoparticles, synthesis, applications

## Abstract

In the last decade, research on the synthesis and characterization of nanosized ferrites has highly increased and a wide range of new applications for these materials have been identified. The ability to tailor the structure, chemical, optical, magnetic, and electrical properties of ferrites by selecting the synthesis parameters further enhanced their widespread use. The paper reviews the synthesis methods and applications of MFe_2_O_4_ (M = Co, Cu, Mn, Ni, Zn) nanoparticles, with emphasis on the advantages and disadvantages of each synthesis route and main applications. Along with the conventional methods like sol-gel, thermal decomposition, combustion, co-precipitation, hydrothermal, and solid-state synthesis, several unconventional methods, like sonochemical, microwave assisted combustion, spray pyrolysis, spray drying, laser pyrolysis, microemulsion, reverse micelle, and biosynthesis, are also presented. MFe_2_O_4_ (M = Co, Cu, Mn, Ni, Zn) nanosized ferrites present good magnetic (high coercivity, high anisotropy, high Curie temperature, moderate saturation magnetization), electrical (high electrical resistance, low eddy current losses), mechanical (significant mechanical hardness), and chemical (chemical stability, rich redox chemistry) properties that make them suitable for potential applications in the field of magnetic and dielectric materials, photoluminescence, catalysis, photocatalysis, water decontamination, pigments, corrosion protection, sensors, antimicrobial agents, and biomedicine.

## 1. Introduction

Nanocrystalline magnetic materials have attracted considerable interest due to their uniqueness and remarkable properties in various fields including physics, chemistry, biology, medicine, materials science, and engineering. Compared to their bulk counterparts, nanomaterials have particle size in the 1–100 nm range and a high surface to volume ratio that determines different or enhanced reactivity, thermal, mechanical, optical, electrical, and magnetic properties [1]. While in the case of bulk materials, the chemical composition is the main factor that determine their properties, in the case of nanomaterials, besides the chemical composition, the particle size and morphology determine most of their characteristics [1,2]. Moreover, these properties can be tunned based on the particle size and chemical composition [1,2].

Ferrites are important and interesting materials, from a practical as well as fundamental point of view. Among various ferrites, nanosized CoFe_2_O_4_, MnFe_2_O_4_, ZnFe_2_O_4_, NiFe_2_O_4_, and CuFe_2_O_4_ have attracted considerable attention due to their chemical and thermal stability and unique structural, optical, magnetic, electrical, and dielectric properties and wide potential technological applications in photoluminescence, photocatalysis, humidity-sensors, biosensors, catalysis, magnetic drug delivery, permanent magnets, magnetic refrigeration, magnetic liquids, microwave absorbers, water decontamination, ceramics pigment, corrosion protection, antimicrobial agents, and biomedicine (hyperthermia) [1,3,4,5,6,7,8].

CoFe_2_O_4_ has an inverse spinel structure with Co^2+^ ions mainly placed in octahedral (B) sites and Fe^3+^ ions almost equally distributed between tetrahedral (A) and octahedral (B) sites. It presents large coercivity (*H*_C_), high magnetocrystalline anisotropy constant (*K*), high Curie temperature (*T*_C_), moderate saturation magnetization (*M*_S_), low remanent magnetization (*M*_R_), excellent chemical and mechanical stability, rich redox chemistry, large magnetostrictive coefficient (*λ*), high electrical resistance, low eddy current losses, significant mechanical hardness, and low toxicity [7,9,10,11,12,13]. The synthesis of CoFe_2_O_4_ with different structures (i.e., nanoparticles, hollow mesoporous nanospheres, nanorods and three-dimensional ordered macroporous structure) have been reported [9,10,13]. Beside many advantages, CoFe_2_O_4_ owns poor electrical conductivity, poor cyclic stability, structural strain, and large volume expansion [9,10,14]. 

ZnFe_2_O_4_ has a normal spinel structure, where Zn^2+^ ions preferably occupy the tetrahedral (A) sites and Fe^3+^ ions the octahedral (B) sites [6,15,16]. The absence of Fe^3+^ in the A sites results in weak antiferromagnetic exchange interactions within Fe^3+^ in B sites, making ZnFe_2_O_4_ antiferromagnetic below 9 K [15,16,17]. The enhanced magnetization originates from the super-exchange interaction ascribed to the inversion of Fe^3+^ and Zn^2+^ ions in the tetrahedral (A) and octahedral (B) sites [18]. 

NiFe_2_O_4_ possesses an inverse spinel structure, where the tetrahedral (A) sites are occupied by Fe^3+^ ions, while the octahedral (B) sites by Fe^3+^ and Ni^2+^ ions, the Fe^3+^ ions being easily distributed between the A and B sites [19,20]. NiFe_2_O_4_ is one of the most versatile and technologically important soft ferrite materials due to its low electrical conductivity, high electrochemical stability, catalytic behavior, abundance in nature, low *K* and *H*_C_ values, high *M*_S_, paramagnetic, superparamagnetic or ferrimagnetic behavior depending on the particle size and shape, low eddy current loss and conductivity, high electrical resistance, and electrochemical stability [4,5,6,11,21]. 

CuFe_2_O_4_ has an inverse spinel structure with 8 Cu^2+^ ions on octahedral sites and 16 Fe^3+^ ions equally distributed between the tetrahedral (A) and octahedral (B) sites [22,23]. The Cu^2+^ ion displays the transition from tetragonal phase, technologically less important (at low temperature) to cubic phase, which is relatively more useful (at high temperature) [24,25]. Moreover, magnetic and electrical properties of CuFe_2_O_4_ vary significantly with the change of cation distribution [25]. CuFe_2_O_4_ exhibits ferrimagnetism, high thermal stability, high resistance to corrosion, excellent catalytic properties, and sufficient band gap, acting as an efficient photocatalyst [22,23,24,26,27].

Manganese ferrite (MnFe_2_O_4_) has a partially inverse spinel structure, fine structural, magnetic and electrical properties, with 20% of Mn^2+^ ions located at octahedral (B) sites and 80% located at tetrahedral sites [3,8]. MnFe_2_O_4_ nanoparticles (NPs) has attracted attention in biomedicine due of its good biocompatibility, controllable size, high magnetization value, superparamagnetic nature and ability to be monitored by an external magnetic field. Moreover, MnFe_2_O_4_ is an inorganic heat-resistant, non-corrosive, environmentally friendly, non-toxic, high shock resistant [28], reusable adsorbent, often employed for adsorption and desorption processes [29] and magnetic drug targeting/delivery [3].

The surface coating of magnetic NPs is required in order to generate non-toxic, biocompatible and water dispersible NPs, along with drug targeting ability. The most used surface coating materials are poly(vinylalcohol), poly(N—isopropylacrylamide), polyethylene glycol (PEG), chitosan, Au, ZrO_2_, and SiO_2_ [3]. Of these, due to the excellent drug loading capacity, stability, non-toxicity, biocompatibility, and water dispersibility, the mesoporous SiO_2_ is a multipurpose candidate for the development of nanocarriers, as it enhances the stabilization of ferrite NPs in water, improves the chemical stability, and minimizes the agglomeration of NPs, without influencing their magnetic and dielectric properties [30]. Non-magnetic SiO_2_ can easily promote conjugation with many functional groups, thus allowing selective and specific coupling and labeling of biotargets. Moreover, SiO_2_ coatings may change the surface properties of magnetic NPs and offer a chemically inert layer, which is particularly useful in biological systems [30,31].

**Figure 1:**
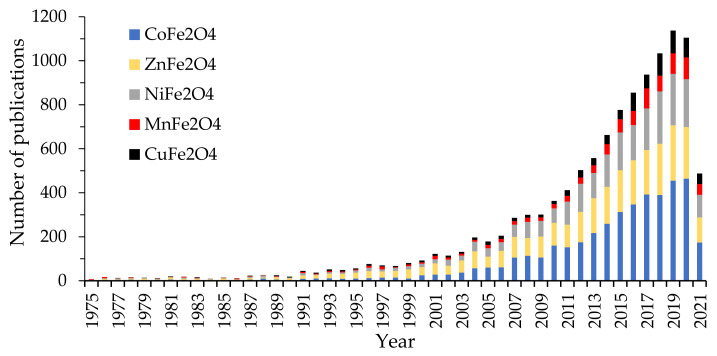
Papers having M-ferrite (M = Co, Cu, Ni, Mn, Zn) in the topic published in Web of Science Core Collection between 1975–June 2021.

By searching in the Web of Science Core Collection the keyword “M ferrite”, where M = cobalt, copper, nickel, manganese, and zinc, we observed that Co ferrite attracted the attention, with the number of publications on Co-ferrite exceeding by far those published on the other ferrites. Between the studied ferrite, Co-ferrite was the topic of 4276 papers, followed by Zn-ferrite (3073), Ni-ferrite (2432), Mn-ferrite (895), and Cu-ferrite (880). For every ferrite, the number of papers started to grow exponentially in the last 20 years (Figure 1), the highest increasing rate being observed for the Co-ferrite. The increasing interest in these ferrites may be attributed both to the development of new equipment that allowed the ferrites characterization and the increase of the demand for materials with special properties for a wide range of applications.

In the last few years, the number of papers that review data on nanosized ferrites with a focus on different ferrites, different synthesis methods, or different applications significantly increased. The Web of Science Core Collection have indexed 58 publication that contains the words “ferrite” and “review” in their title of which 25 were published between 2019 and June 2021. 

Zate et al. [32] reviews the mechanical, chemical, spray and electrospining methods used for the synthesis of different ferrites with magnetic properties. Vedrtnam et al. [33] reviews the properties, classification, synthesis, and characterization of hexagonal and spinel ferrites with a focus on the main four synthesis routes (sol-gel, hydrothermal, co-precipitation and solid-state), magnetic properties, and characterization of the ferrites. Vinosha et al. [34] review the recent advances of synthesis, magnetic properties, and water treatment applications of cobalt ferrite, while Masunga et al. [35] reviews the recent advances in copper ferrite synthesis, magnetic properties and application in water treatment. Kumar et al. [36] review magnetic nano ferrites and their composites used in the treatment of pollutants from waste waters, emphasizing pros and cons of several synthetic pathways, the adsorption mechanism, and ferrite regeneration, while Kefeni and Mamba [37] review the photocatalytic application of spinel ferrite NPs in pollutant degradation with emphasis on the possible recovery and reuse of NPs. Kharisov et al. [38] review the use of cobalt, nickel, copper, and zinc ferrites and their doped derivates as catalysts in organic processes, while Dalawai et al. [39] overview the spinel-type ferrite thick films together with preparation strategies and sensors, microwave, magnetic, and advanced applications. Kefeni et al. [40] review the ferrite’s applications in electronic devices, such as sensors and biosensors, microwave devices, energy storage, electromagnetic interference shielding, and high-density recording media together with the advantages and drawbacks of most important ferrite NPs synthesis methods. 

Our previous works reported the thermal, structural, morphological, and magnetic characterization of ferrites (MFe_2_O_4_, M = Co, Mn, Zn, Ni and Cu) and doped ferrites with different divalent transition metals, produced by sol-gel synthesis [41,42,43,44,45,46]. Furthermore, since the physical, chemical, magnetic, electrical, and optical properties can be tailored by the dopant type and content, we also reviewed the potential applications of CoFe_2_O_4_ and divalent transition metal-doped cobalt ferrites (M_x_Co_1−x_Fe_2_O_4_, M = Zn, Cu, Mn, Ni, and Cd) [47]. In addition, this paper aims to deepen our previous studies and review a number of topics including the various synthesis methods of CoFe_2_O_4_, MnFe_2_O_4_, ZnFe_2_O_4_, NiFe_2_O_4_, and CuFe_2_O_4_ NPs together with their advantages and disadvantages, and the most important applications in conventional and modern technologies. The review not only summarizes the existing literature from theoretical and methodological points of view, but also synthetizes it from a new perspective, representing a significant and useful contribution to subsequent research.

## 2. Synthesis Methods

**Figure 2:**
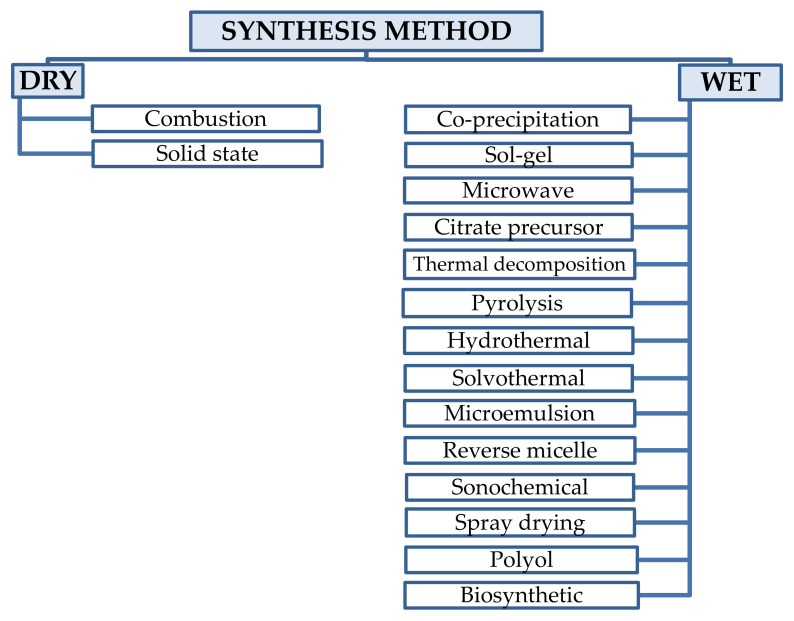
Classification of nanoferrite synthesis methods.

The chemical and physical properties of transition metal nanoferrites (MFe_2_O_4_; M = Co, Ni, Zn, Mn, Cu) are dependent on the synthesis method and conditions. Thus, the selection of an appropriate synthesis route plays a crucial role in tailoring the properties and obtaining high quality nanoferrites [1]. Nanosized ferrites can be synthesized by various techniques (Figure 2) and the possibility to obtain almost any solid solution of nanoferrites unlocks the way to tailor their properties for a wide range of applications [22]. 

There are different classification approaches of the ferrite synthesis methods: (i) physical, chemical, and biological, based on the type of processes that take place in the synthesis methods, (ii) dry and wet methods, based on the presence or absence of a solution, and (iii) conventional and non-conventional, based on their novelty. Despite several classification methods, it is difficult to univocally group the synthesis methods, as sometimes different processes are coupled to obtain NPs with specific characteristics.

Many efforts have been made to tailor the size, shape, particle size distribution, surface area, composition, structure, and properties of the ferrite NPs by employing different synthesis methods, or changing the synthesis parameters, such as the annealing temperature and duration, concentration of reactants, pH value, stirring speed, doping additives, etc. [48,49]. Numerous physical and chemical methods have been used for the synthesis of ferrites [1,19,25]. Some synthesis methods are high-energy consuming, complex procedures, demanding a high processing temperature and long reaction time to complete the crystallization, and the use of reduction agents with negative effects on the environment [6,13]. The importance of these factors is different in every method, making the achievement of reproducibility in desired properties difficult [50]. Another issue that appears in the majority of the synthesis methods is the agglomeration of NPs after production, which limits the control of size, shape and function [51]. The wet-chemical synthesis has multiple advantages. However, single phase ferrite can be obtained only after annealing at high temperatures and is accompanied by particle growth, aggregation, and coarseness of NPs [14,20]. 

### 2.1. Dry synthesis Methods

#### 2.1.1. Combustion Method 

Combustion method is simple, fast, and inexpensive, as it does not involve intermediate decomposition or calcinations steps. This method exploits an exothermic, usually very rapid, and self-sustaining chemical reaction between metal salts and an organic fuel (glycine, urea, citric acid, sucrose, hydrazine, and polyvinyl alcohol) that act as a reducing agent [14,52]. The powder characteristics (i.e., crystallite size, surface area) are governed by the enthalpy or flame temperature produced during the combustion, which is dependent on the type of fuel and fuel-to-oxidant ratio. Beside the key role of fuel in the morphology of the NPs it also determines the phase formation [53]. An important aspect is that the heat necessary to sustain the chemical reaction is provided by the reaction itself and not by an external source [8]. Nitrates are the favorite metal precursors because of their high solubility in water and easy combustion following their mixing with a suitable fuel [7,54]. Ammonium nitrate is used as an extra oxidant in the combustion reaction, producing the expansion of the microstructures and eventually the increase of NPs surface area, without changing the proportion of the other participants [7]. The combustion method is a very popular method for the synthesis of ceramic materials, composites, and ferrimagnetic nanomaterials, due to its efficiency, short preparation time, use of relatively inexpensive precursors and simple equipment, good control of stoichiometry, desired particle size distribution, formation of high-purity products, and stabilization of metastable phases. The main disadvantages are high combustion temperature and low production yields [6,11,13,14,55]. The combustion method is a good choice for the preparation of high quality CoFe_2_O_4_, NiFe_2_O_4_ and MnFe_2_O_4_ NPs. By varying the nitrates to fuel ratio, the size, *M*_S_ and *H*_C_ values are tailored [20,56,57]. Nanocrystalline NiFe_2_O_4_ was successfully prepared by mixing of metal nitrates and citrate with the formation of a colloidal solution (sol), followed by the continuous heating of xerogel, the auto combustion process until the formation of a loose powder, and the annealing of the powder at 700 °C [21]. This method requires less time and produces pure and homogeneous NPs, without any type of waste product, but involves a high temperature [7,58,59]. The high purity of materials prepared by the combustion process is attributed to the removal of unwanted impurities as volatile species at high temperatures [6].

#### 2.1.2. Solid State Synthesis 

Solid state synthesis produces polycrystalline ferrite nanomaterials from solid reagents at high temperature [20]. This method assumes the grinding and mixing of metal nitrates or sulphates with NaOH or NaCl in an agate mortar or mills for short times. After the removal of NaCl by washing, the powders are dried at 80 °C for two hours and then annealed at 700 °C for two hours [60]. CoFe_2_O_4_ NPs have been synthesized by a low-temperature solid state process using various salts (sulphate, acetate, nitrate and chloride). By using chloride salts, the obtained nanomaterials have smaller particle size than by using other salts [61]. Furthermore, in comparison to other synthesis techniques, the low temperature solid state process requires neither complex apparatus, nor solvent or solution, and is a convenient, environment-friendly, low-cost, time-saving and low energy consumption process [62]. The CoFe_2_O_4_ NPs prepared using solid state technique display high *M*_S_ values, as well as low *M*_R_ and low *H*_C_ values [63]. The major advantages are that the synthesis procedure is completed under atmospheric pressure, which is economic and feasible, as well as the use of non-expensive and toxic solvents and cost-effective raw materials [58]. Mechanical milling is a simple, low-cost, solid state and non-equilibrium process in which the final product have a very fine, typically nanocrystalline or amorphous structure. Generally, the final product has a nanosized structure with enhanced properties and performance compared to the bulk material [15]. The main advantages of this method are simplicity, low cost and ability to produce large volumes [63]. Mechanical milling of appropriate metal oxides for 12 h and subsequent sintering at 600 °C for 2 h were applied to prepare ZnFe_2_O_4_ and NiFe_2_O_4_ ferrites [15]. CoFe_2_O_4_ NPs were obtained by ball milling of Fe_2_O_3_ and Co_3_O_4_ powders in stoichiometric amounts using high-energy vibratory mill for 8 h, followed by calcination at 900 °C, for 12 h [64]. CoFe_2_O_4_ NPs of 10 nm size were also prepared by the precipitation of hydroxide/oxidhydroxide, followed by mechanical milling at lower speeds, and subsequent heat treatment for a short time. NaCl was added before milling to avoid agglomeration [62]. NiFe_2_O_4_ crystallites were prepared by high energy ball milling. The increase of milling time led to a significant increase in the NiFe_2_O_4_ formation and to a progressive decrease in its particle size and lattice parameter [20]. MnFe_2_O_4_ NPs (4–8 nm) were prepared by solid state ball-milling and calcinations (300–400 °C) of nitrate precursors and citric acid [65]. 

### 2.2. Wet Synthesis Method

#### 2.2.1. Co-Precipitation Method

Co-precipitation method is one of the most widely used methods due to its high yield and simplicity in producing high purity ultrafine magnetic nanostructured ferrites [58,66]. The co-precipitation method is a very simple and cost-effective method that allows an easy control of particle size and composition, requires low temperature and leads to materials with high crystallinity, homogeneity and good textural properties [1,58]. The major drawbacks of co-precipitation methods are extensive agglomeration, poor crystallinity and particle size distribution, and the necessity of pH control [57,67].

In this case, homogeneous solutions are formed by dissolving inorganic salts (chloride, sulfate, nitrate) in water or solvents. After pH adjustment in the range of 7–12 under continuous stirring, the precipitate is collected by filtration or centrifugation, washed, and dried. The pH change rate causes the particles aggregation and crystal growth [58]. The most common way to synthesize nanostructured ferrites by chemical co-precipitation method is using Co^2+^ and Fe^3+^ salts in the presence of a strong base [51]. By adjusting the experimental parameters (i.e. reaction temperature and time, reagents feeding rate and concentration, pH, drying temperature, etc.), the size, shape, and magnetic properties of the nanostructures may be controlled [68].

CoFe_2_O_4_ NPs (2–47 nm) were synthesized from metal chloride salts using different concentration of aqueous NaOH and NH_4_OH solution using a reaction time of 2 h and variable reaction temperature (20–100 °C). To achieve the precipitation reaction, the aqueous metal chloride solutions were slowly added into the preheated boiling aqueous alkaline solution [68]. The crystallite size depends on the reaction temperature, time, concentration of the base solution, and pH [68]. Another study reported the preparation of CoFe_2_O_4_ NPs (2–14 nm) by controlling the co-precipitation temperature of Co^2+^ and Fe^3+^ ions in alkaline solution from 20 to 80 °C [49]. 

The co-precipitation of CoFe_2_O_4_ nanocrystals by mixing Fe^2+^, Co^2+^ and NaOH in the presence of oxidizing agent such as KNO_3_ to convert Fe^2+^ into Fe^3+^ was reported by Chia et al. [66] and Senapati et al. [69]. However, due to the coexistence of both Fe^2+^ and Fe^3+^ ions, besides CoFe_2_O_4_, secondary phase such as magnetite (Fe_3_O_4_) is also formed [66]. Moreover, the *H*_C_ values of particles obtained by this procedure are not very high and depends on the amount of surfactant used, that favor a stable colloidal dispersion of the NPs, as well as on the annealing temperature [69].

A detailed investigation on the effect of precursors, solvents, precipitating agent, and heat treatments on the particle size, particle size distribution, morphology, and chemical composition of CoFe_2_O_4_ NPs synthesized at various liquid phase was reported by Prabhakaran et al. [68]. In some cases, in order to facilitate the solubilization of metal salts at low temperatures or to prevent oxidation and particle agglomeration, some surfactants such as oleic acid are added to the solution before precipitation [1].

The temperature may influence the size and stability of the NPs [50]. The particle size growth in the co-precipitation method is influenced by the difference between local and surface temperature, as the formation of spinel nano-ferrites is usually an exothermic reaction, with the latent heat being released at the surface [70]. At low temperatures particles tend to aggregate, the particle size increasing with the decrease of temperature. High purity and no aggregation of the NPs were reported at high temperatures due to directed crystallization [50].

Reverse co-precipitation is similar to conventional co-precipitation, except that instead of adding a precipitant into the precursor ions solution, the precursor ions are added into the precipitant solution. Thereby, the precipitant is in a supersaturated state, ensuring the complete precipitation. The resultant NPs have smaller particle-size than those obtained by traditional co-precipitation [71]. The synthesis of CoFe_2_O_4_ NPs by reverse co-precipitation was reported by Huixia et al. [58].

#### 2.2.2. Sol-Gel Method 

The sol-gel method is a low temperature process based on hydrolysis and condensation reactions of metal precursors (salts or alkoxides), leading to the formation of a three-dimensional inorganic network [72]. Sol results from the conversion of monomers into a colloidal solution, while gel is obtained after the solvent evaporation by joining together particles into a network [58]. The sol-gel method is a simple, low cost, and environmentally friendly method to prepare nanocomposites (NCs) as it allows good control of the microstructure, particle size, dispersion, structure, and chemical composition by carefully monitoring the preparation parameters [58,73,74]. The sol-gel method has been used to prepare very fine, highly dense, homogenous, and single-phase ferrite NPs. Compared to other conventional methods, the sol-gel displays a good stoichiometric control and also allows the production of ferrites at relatively low temperature [20]. The obtained nanomaterials may be formed either as films or as colloidal powders [58]. The main disadvantage is the limited efficiency and long duration of the synthesis process [32,48,58]. NiFe_2_O_4_ nanostructures with an average particle size of 27 nm were synthesized by sol-gel method using glycolic acid as a chelating agent [20]. In the case of CoFe_2_O_4_@SiO_2_, the SiO_2_ network protects the NPs and minimizes the surface roughness and spin disorder. The *H*_C_ values at room temperature (RT) for CoFe_2_O_4_@SiO_2_ were much higher than that of unembedded CoFe_2_O_4_ [73]. The most commonly used reagents are metal nitrates as metals source, 1,2-ethanediol, 1,3-propanediol or 1,4-butanediol as chelators, tetraethyl orthosilicate (TEOS) as matrix precursor, ethanol as solvent and HNO_3_ [32]. By annealing, the NPs agglomerate and form larger particles. Thus, further research on the influence of thermal energy released during heat treatment on the undesired growth of particle size is needed [70]. 

The Pechini method is an alternative approach to sol-gel synthesis and consists in the complexing of cations with hydroxycarboxylic acids (usually citric acid or ethylenediaminetetraacetate) in an aqueous-organic medium. The obtained chelates are cross-linked and transformed into polymers using polyalcohol (ethylenediol, polyvinyl alcohol) through polyesterification. By heating, the viscous liquid is dried and forms a gel. By annealing the gel, the organic part is removed, resulting in reactive oxides, which further form the ferrites [75,76,77]. The microwave-assisted Pechini method combines the advantages of microwave and Pechini methods, such as low temperature and time of process, accurate control of stoichiometry, uniform mixing of various components on molecular level, low price of precursor and equipment, and capability of industrial scale-up [31,75]. However, the main drawback of this method is the long and energy-wasting thermal treatments in order to remove the large amounts of organic precursors [75]. The microwave-assisted sol-gel Pechini method is a faster, energy-saving procedure for obtaining single-phase nanopowders of high purity [75].

Combining the sol-gel and auto-combustion methods results in a simple and inexpensive preparation method for high purity, homogenous nanopowders at low annealing temperature [56,61]. The method consists in dissolving nitrate salts in water, addition of the organic complexing agent (citric acid), adjustment of the solution to pH 7, heating at 70 °C to form the sol, and then at 110 °C to remove the residual water and form the gel, and initiation of the autocatalytic self-combustion process of nitrate-fuel gel [78]. The citric acid and glucose are two of the most used fuels due to their strong complexing ability and low ignification temperature (200–250 °C) [78]. The synthesis of CoFe_2_O_4_ NPs in the size range of 11–40 nm by the sol-gel auto combustion method and annealing at different temperatures (800–1000 °C) was also reported [78]. 

The organic precursor method is similar to the Pechini method and involves the preparation of aqueous solution containing cations, chelation of cations using carboxylic acid, followed by heating of the solution until precursor formation and annealing of the precursors. The carboxylic acid acts both as complexing agent and as organic rich fuel. Magnetic CoFe_2_O_4_ NPs (38.0–92.6 nm) were produced by the organic precursor method using oxalic and tartaric acids as precursors [79].

#### 2.2.3. Microwave-Assisted Combustion Method 

The microwave-assisted combustion method applies microwave radiations to the reaction solution to synthesize NPs. Microwave radiation is absorbed and converted to thermal energy, the heat being generated inside a material, unlike the conventional heating methods where the heat is transferred from the outside. This heating allows for a considerable reduction of processing time and energy [13,54,80]. The main advantages of this method compared to other synthesis methods are simplicity, homogeneous nucleation, rapid reaction time, high production rates, environmentally friendly, excellent reproducibility, low energy cost, easy handling, and control of parameters. In the microwave-assisted combustion process, the reagents are mixed at the molecular level, due to the interaction of microwaves, offering an excellent control of stoichiometry, purity, homogeneity, and morphology [53,58,59]. However, the method is expensive mainly due to the expensive fuels such as urea, glycine, L-alanine, carbohydrazide, or citric acid used to promote and control the combustion process in accordance with the propellant chemistry principles, and inappropriate for scale up and reaction monitoring [13,53,80,81,82]. 

In the case of the microwave hydrothermal method, the necessary heat is generated by microwaves, which have the advantage of very fast heating to a certain depth, allowing the generation of homogenous nanomaterials with fine particle size distribution [52,53]. Zn, Fe, Mn, Cu, Ni, and Co nitrates were dissolved in deionized water, maintaining a pH equal to 9.4. The mixture was sealed with tetrafluorometoxil (TFM) and placed in a microwave oven at 160 °C for 30 min. The resulting wet mixture was dehydrated, followed by adding polyvinyl alcohol (PVA) as a binder and sintering for 30 min [58]. This method provides faster heating, is more economical, and produces very fine, uniform NPs [59].

#### 2.2.4. Citrate Precursor Method 

The citrate precursor method is a wet chemical process that involves the mixing of aqueous solutions of precursor salts (metal nitrates) with an aqueous solution of citric acid, followed by heating at about 80 °C and annealing at 700 °C [83,84]. During this process, the precursors are thermally decomposed into ferrite powders [83]. The advantage of this method is the high reactivity, low reaction time, low synthesis temperature, homogenous distributions of ions, and low cost over other chemical methods [83]. To reduce the particle agglomeration, the dispersion of NPs in SiO_2_ matrix is a commonly used. The synthesis of CoFe_2_O_4_@SiO_2_ NPs by citrate method using TEOS, ethanol, Fe and Co nitrates, citric acid, and ethylene glycol was successfully reported by Garcia [83] and Varma [62], with the latter reporting 50 nm size and *M*_S_ values in the range of 4–25 emu/g.

#### 2.2.5. Thermal Decomposition Method 

The thermal decomposition method is based on the heating of reactants (metallic precursors) at different temperatures. To control the nucleation and growth of the NPs and consequently improve the physical properties of the material, such as crystallite size, porosity, and specific surface, surfactants (oleylamine, oleic acid) are added in the decomposition step. The surfactants act as a protective envelope coating the NPs, limiting the coalescence, and leading to the improvement of the physical properties of the material, such as crystallite size, porosity, and specific surface. These parameters allow the control of the magnetic properties [85]. 

The method is relatively simple, low-cost, takes place at low reaction temperature, is environment friendly, produces highly monodispersed particles with a narrow size distribution, and does not produces byproducts. The main drawback is the removal of surfactants simultaneously with the particle size controlling [50,86]. The use of thermal treatment to synthesize ferrite NPs is very limited, although this method requires reduced time as compared to other synthesis techniques [87]. The thermal decomposition method, reflux temperature, and time play an important role in controlling size distribution and aggregation of CoFe_2_O_4_ NPs, which will significantly influence the magnetic properties such as permeability and coercivity of the products. The reaction rate depends on the concentration of metal precursors, surfactants, reducing agents and temperature [88]. One example of thermal decomposition is the dissolution of metal nitrates in deionized water, followed by their dispersion in a solution of polyvinylpyrrolidone (PVP) at 70 °C, followed by a thermal treatment at 600 °C for four hours [87]. Asghar et al. [3] reported the synthesis of MnFe_2_O_4_ by dissolving Fe and Mn acetates (2:1, molar ratio) in dibenzyl ether, in the presence of 1,2-dodecanediol, oleic acid, and oleylamine, followed by a thermal treatment at 250 and 350 °C, respectively. Peddis et al. reported the synthesis of crystalline CoFe_2_O_4_ NPs coated by oleic acid and organized in a self-assembling arrangement with narrow size distribution (∼5 nm) by high thermal decomposition using Fe(III) acetylacetonate, Co(II) acetylacetonate, 1,2-hexadecanediol, oleylamine, oleic acid, and phenyl ether as solvents, heated to 200 °C for 30 min and to 265 °C for 30 min [89]. Following the thermal treatment method, in aqueous solution containing metal nitrates and polyvinyl pyrrolidone, followed by grinding and calcination at temperatures ranging between 723 and 873 K, MnFe_2_O_4_ NPs (12–22 nm) were produced [86].

#### 2.2.6. Pyrolysis

##### Spray Pyrolysis Method

The spray pyrolysis method consists in converting the reagent mixture into aerosol droplets, solvent evaporation, solute condensation, and drying followed by thermolysis of the particles at high temperature [50]. This method allows the control of the particle formation environment by dividing the solution into droplets [90]. Generally, it is suitable for the synthesis of mixed metal ferrites as it ensures complete stoichiometry retention on the droplet scale and provides a highly homogeneous distribution of the components [90]. By controlling the type of thermolysis reaction, the type of precursors, the gaseous carrier, deposition time, and substrate temperature, this method allows the synthesis of a broad range of hollow or porous particles with potential applications in thermal insulation or catalyst support [90,91]. 

Non-agglomerated particles smaller than 10 nm are achieved by using soluble inert additives (NaCl, H_3_BO_3_) to the reaction mixture. Furthermore, the inert components allow subsequent thermal treating of the obtained powder without significant particle size growth [89]. The method’s advantages include obtaining predictably sized, finely dispersed particles of variable shape and composition, high production rate, scalability, and reduced time [50,89]. The disadvantages of the method are the high costs and possibility to obtain aggregated particles [50]. Zn_x_Fe_3-x_O_4_ and CoFe_2_O_4_ thin films were produced by spray pyrolysis technique, by spraying aqueous solution of metal nitrates at a temperature of 300 °C. After deposition, film was naturally cooled [92]. CoFe_2_O_4_ thin film is prepared by the spray pyrolysis technique due to its easy handling, simple experimental setup, and cost effectiveness as large surfaces of thin film can be produced [92].

##### Laser Pyrolysis 

Laser pyrolysis is another method for the synthesis of nanosized ferrites. The gaseous phase precursors are transported by a carrier gas introduced into a reaction chamber where a high-power laser beam (2400 W) generates elevated local temperatures, which trigger the nucleation and growth of NPs that are further collected by a filter [93]. Liquid reactants are introduced into the reaction chamber reactants as vapors or microscale droplets [94]. This technique allows the production of high purity nanomaterials with small and narrow particle size distribution in one step. The method is versatile and flexible, and allows the control of operational parameters, such as laser power, type of gases, gas flow rate, and concentration of the precursors [94]. Moreover, it can be easily scaled-up to industrial production. The disadvantages of the method include the high cost of equipment used and the need for a precursor with absorption band at 10.6 μm, or addition of C_2_H_4_ or NH_3_, which leads to further contamination with carbon or nitrogen [93,94]. The synthesis of ZnFe_2_O_4_ nanopowders by laser pyrolysis using Fe and Zn nitrate solutions as precursors, ethylene as sensitizer gas and air as carrier gas was reported. The obtained nanopowders were tested as negative electrode materials for Li-ion batteries [94]. 

#### 2.2.7. Hydrothermal Method 

The hydrothermal method is used for the preparation of ferrite NPs at large scale owing to its high yields [58,59]. The method consists in the NPs formation by mixing the aqueous divalent metal (Ni, Co, Mn, Zn, Mn) acetate solutions with iron nitrate and a carbon-based nano template at alkaline pH and its dispersion around 200 °C in an autoclave. The obtained precipitate is washed, separated by centrifugation, and annealed around 500 °C to eliminate the carbon-based template [95]. In order to control the crystal structure, particle size, and morphology, surfactants are often employed in solution process [96]. Ethylenediamine and citric acid may also be used in the synthesis. This method requires neither sophisticated processing nor high processing temperature and allows the selection of the NPs properties by using different temperatures, pressure, reaction times, and nano templates [59,97].

Advantages of the hydrothermal method are good nucleation control, production of low particle size with narrow particle size distribution, controlled morphology at a high reaction rate and different temperature and pressure levels, high yield, excellent stoichiometry, and simplicity [67]. 

The production of magnetic nanosized CoFe_2_O_4_ by hydrothermal method without subsequent calcination processes was reported [98]. However, the influence of the synthesis time on the morphology and the particle size of CoFe_2_O_4_ NPs is still under discussion [96]. CoFe_2_O_4_ nanorods using cetyltrimethylammonium bromide (CTAB) as the surfactant were also synthesized by the hydrothermal method [99]. Porous anodic aluminum oxide is also used as template in the synthesis of one-dimensional CoFe_2_O_4_ nanostructures, however the costs of the synthesis are high while the yields are low [99]. The decrease of the reaction medium pH favors the CoFe_2_O_4_ crystallite size decrease. However, pure CoFe_2_O_4_ at pH < 10 was not obtained independently of the temperature and reaction time [100]. CoFe_2_O_4_ nanoplatelets and NPs were also prepared by hydrothermal reaction from FeCl_3_, cobalt dodecyl sulfate and NaOH aqueous solution, in strictly controlled synthesis environment [101]. CoFe_2_O_4_ NPs were also synthesized by the supercritical hydrothermal method at different temperatures of 25–390 °C for two hours [58,59,68,97]. 

NiFe_2_O_4_ nanocrystals were synthesized from FeCl_3_·6H_2_O and NiCl_2_ 6H₂O, using cetyltrimethylammonium bromide (CTAB) as the surfactant and NH_3_ and NaOH as hydrolyzing agents [101]. Moreover, ZnFe_2_O_4_ were synthesized using metallic Zn sheet and FeCl_2_ as reactants in ammonia solutions [101]. Hydrothermal synthesis of MFe_2_O_4_ (M = Ni, Co, Mn, Zn) using metal acetylacetonate and aloe vera extracts provided high-yield nanosized ferrite with well crystalline structure using high calcination temperature [101]. 

#### 2.2.8. Solvothermal Method 

The solvothermal method has emerged as a promising technique to synthesize monodisperse spherical magnetic microspheres with high surface area, magnetic saturation and good dispersion in liquid media [102]. CoFe_2_O_4_ magnetic NPs in the size range 2–15 nm were prepared using a non-aqueous solvothermal method by dissolving Co(III) acetylacetonate and Fe(III) acetylacetonate in acetophenone, followed by solvothermal treatment between 120 and 200 °C for 22 h in a Parr Acid Digestion Bomb autoclave [103]. The solvothermal method was successfully applied to synthetize CoFe_2_O_4_, NiFe_2_O_4_, MnFe_2_O_4_, and ZnFe_2_O_4_ using sodium or ammonium acetate, polyethyleneglicol, urea, and oleylamine at around 200 °C [102]. Size-controlled NiFe_2_O_4_ NPs were successfully synthesized via a simple solvothermal method using ethylene glycol as solvent and sodium acetate as electrostatic stabilization, by adjusting the experimental parameters (time, initial concentration of reactants, amount of protective reagents, and the type of acetates) [20]. The main advantages of the method are high performance in biological applications by obtaining magnetic NPs with smooth surfaces, narrow size distributions, large surface areas, and high magnetic saturation in order to provide maximum signal and good dispersion in liquid media [49].

#### 2.2.9. Microemulsion Method 

The microemulsion method consists in the mixing of microemulsions containing reactants, the formation of micro droplets, followed by the trapping of fine aqueous micro droplets inside surfactant molecule assembles. This procedure results in a locking up effect that limits the growth dynamics, particle nucleation, and agglomeration during the nanoparticle formation. The major advantage is the obtaining of monodispersed NPs with various morphologies, while among the major disadvantages can be listed the low efficiency and difficulty to scale up [69]. CoFe_2_O_4_ NPs lower than 50 nm, with high *H*_C_ and *M*_S_ were synthetized by a simple micro-emulsion synthesis route using water-in-oil emulsions consisting of water, cetyltrimethyl ammonium bromide as surfactant, n-butanol as co-surfactant, and n-octane as oil phase [104]. CoFe_2_O_4_ NPs of 4–25 nm were prepared using normal micelle microemulsion methods [105].

#### 2.2.10. Reverse Micelle Method 

The reverse micelle method is based on the formation of water-in-oil emulsions in which the water to surfactant ratio controls the size of water pools within which aqueous chemical syntheses take place [106]. The method consists in mixing the metal salt precursors and the precipitating agent and the formation of microemulsion droplets, which act as a nano-reactor, in which nanometric size precipitates are formed following the coalescence and droplet collision of reactants [107]. This method allows an excellent control of the particle size, size distribution, chemical stoichiometry, and cation occupancy and is suitable for room temperature reactions such as the precipitation of oxide NPs [106]. 

Metallic chlorides and NaOH were dissolved by ultrasonication in two different microemulsion systems prepared by mixing water, sodium dodecyl sulfate, 1-butanol and n-hexane. The two microemulsions were mixed together till metallic hydroxides were precipitated, the obtained precipitates were filtered, washed, dried and annealed at 400 °C [107]. The nature of surfactant, water to surfactant ratio and pH value of solution strongly influences the particle size of ferrites, which in turn affects their properties [101]. For the synthesis of CoFe_2_O_4_ a surfactant system formed by mixing sodium dioctylsulfosuccinate with a 2,2,4-trimethylpentane oil phase was used [106]. CoFe_2_O_4_ magnetic NPs were synthesized by reverse micelle methods using a micro emulsion consisting of three independent phases, (petroleum oil, pyridine and water) as a template to control the size of the NPs [108]. MnFe_2_O_4_ NPs with particle size of approximately 8–25 nm were prepared using reverse micelle microemulsion methods [105]. Using the reverse micelle microemulsion method, a wide range of spinel ferrite nanoparticle cores can easily be coated with a silica shell [105]. Such a method increases the potential for development of tunable magnetic silica for magnetoelectronic and biomedical applications [105].

#### 2.2.11. Sonochemical Method

The sonochemical method is considered one of the most promising procedures to obtain ferrite NPs. The sonochemistry arises due to acoustic cavitation phenomenon consisting in the formation, growth and collapse of bubbles in liquid medium. The extreme reaction conditions (i.e., high temperature (5000 K), pressure (20 MPa) and cooling rate (1010 K/s)) lead to numerous unique properties of the produced particles [109]. Highly crystalline, monodisperse CoFe_2_O_4_ NPs with uniform spherical shape and high *M*_S_ values were synthetized in a one-step, surfactantless, sonochemical process. Additionally, the synthesis time was low (about 70 min) and no subsequent annealing was necessary [109]. The advantages of this approach over the conventional methods include simplicity, low cost, safety, environment friendly, uniform size distributions, high surface area, fast reaction time, and good phase purity [109]. Disadvantages comprise a very small concentration of prepared NPs and particle agglomeration [50].

#### 2.2.12. Spray Drying Method 

The spray drying method is used for the large-scale production of ZnFe_2_O_4_ and MnFe_2_O_4_. The method is based on the spraying of the ferrite slurry droplets into a hot air vertical evaporating tube. The main drawbacks are the inflation defects visible as large voids inside the granule and the development of capillary stress due to the rapid water evaporation. This stress may lead to undesirable diffusion and segregation phenomena, which reduce the compositional and morphological homogeneity of NPs [110]. As the defects may resist annealing, to preserve the requested particle size and avoid the occurrence of defects, the spray droplets may be frozen using liquid nitrogen and freeze-dried. During water sublimation, there are no undesirable capillary stresses that generate hard unbreakable aggregates and result in voids that lead to a rigid porous product of loose and non-agglomerated particles [110]. 

#### 2.2.13. Polyol Method 

The polyol method consists in the reduction of metallic oxide or metallic complexes by a high boiling point solvent that acts as a solvent as well as a reducing agent of the metallic ions under reflux conditions. The method allows controlling particle growth and preventing the particles agglomeration by the suspension of precursors into liquid polyol (ethylene glycol, diethylene glycol and triethylene glycol), followed by thermal treatment up to boiling point of polyol. The major advantage of this method is the obtaining of uniform size soft and hard magnetic NPs, while the main disadvantages are the high temperature and long time [111,112]. The polyol method has been used for synthesis of many oxides and NCs materials [73]. To produce CoFe_2_O_4_, FeCl_3_·6H_2_O and CoCl_2_·4H_2_O were individually dissolved in glycol, mixed together under continuous stirring. To the homogenous solution, water and sodium acetate were added, and the pH value of the solution was adjusted (8, 10, and 12) by adding ammonia solution. Under reflux conditions, the mixture was heated to the boiling temperature of glycol, while the formed NPs were separated by centrifugation [111]. The synthesis of NiFe_2_O_4_ NCs starting from tetraethoxysilane without, or with modifiers as formamide, citric acid, and PVA were also reported [4].

#### 2.2.14. Biosynthetic Method

The biosynthetic method uses plant extracts as a simple and viable alternative to chemicals during the synthesis process. The use of microorganisms, enzymes, and plant extracts has been proposed as a possible eco-friendly alternative to chemicals of physical synthesis. Furthermore, the biosynthetic route is very simple and provides high-yield, crystalline nanomaterials with adequate properties [13]. Aloe vera (*Aloe barbadensis mill*.) plant-extract may be used as a bio-reducing agent in the preparation of ferrites, providing a simple, efficient and green alternative route for the synthesis of nanomaterials. A possible explanation for this could be the presence of long chain polysaccharides in the Aloe vera plant extract that allows the homogeneous distribution of ferrite [13]. The major advantages consist in the selectivity and precision of NPs formation, cost-effectiveness, as well as the use of an eco-friendly reducing agent and non-hazardous gelling agent for stabilizing the nanostructures. Disadvantages are the difficult size and properties controlling, high temperature required to convert the precursors in crystalline materials and the formation of polydisperse, surface capped or unpurified NPs, in addition to poor reproducibility [13,50]. The use of Ni and Fe nitrates and freshly extracted egg white (ovalbumin) in an aqueous medium, results in the formation of NiFe_2_O_4_ NPs with ferrimagnetic behavior, *M*_S_ values in the range of 26.4–42.5 emu/g for the applied field of 10 kOe, at room temperature [20].

In conclusion, although different synthetic routes have been adjusted, the key challenge in the synthesis field remains the obtaining of size- and phase-controlled synthesis, with good reproducibility. However, reproducibility is difficult to achieve in the synthesis methods where partial mixing of reactants and undesired reactions take place, affecting the properties of NPs. Moreover, the heat and mechanical treatments can alter crystal structure, affecting the photocatalytic activity. The combustion method is simple and quick, but the rate of organic fuel to nitrates can highly affect the size and magnetic properties. The solid-state method at higher temperatures produced micron-sized CoFe_2_O_4_ particles, while the citrate precursor method at lower temperatures produced NPs. To overcome these challenges in an ideal, controlled reproducible synthesis, the following steps should be achieved: (i) direct active and complete mixing of reactants, (ii) automation, and (iii) enabled reaction parameters controlled precisely. Each synthesis method has its own pros and cons, and selection depends on many factors, but amongst all methods of synthesis, the sol-gel and chemical co-precipitation techniques stand to be favorable routes to synthesize homogeneous, highly pure, and narrow size distribution nanoferrites.

## 3. Applications

**Figure 3:**
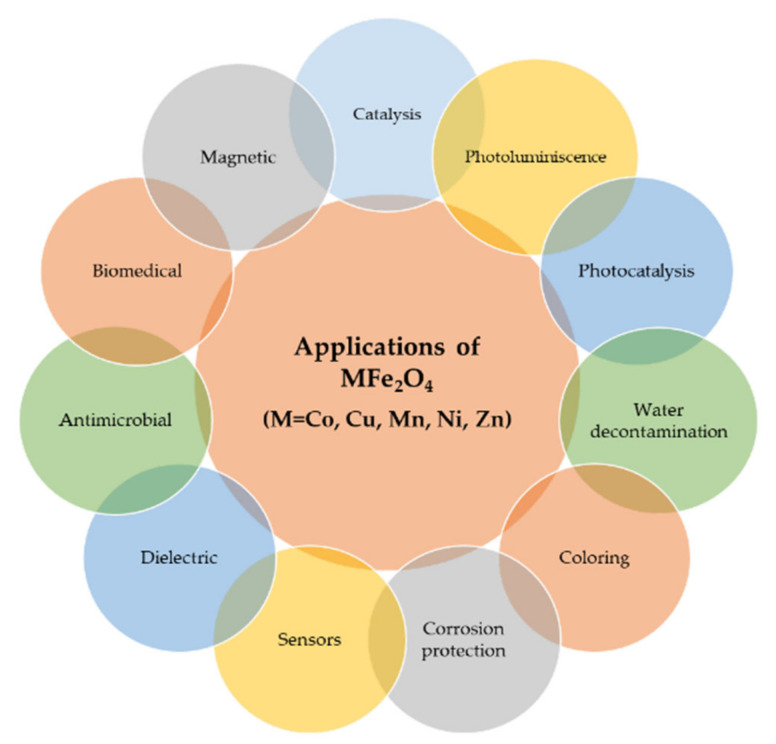
Application of nanosized ferrites.

The most frequent applications of MFe_2_O_4_ (M = Co, Cu, Mn, Ni, Zn) are presented in Figure 3. Considering the cation type and synthesis route, the structure of the ferrite and its particle size and shape changes and thus also its properties, which further lead to different applications. 

### 3.1. Magnetic Applications

The dependence of the magnetization on the grain size is due to the variations of exchange interaction between tetrahedral and octahedral sites and [20,113]. For their application in high-density magnetic recording, the magnetic particles should have nanosize to prevent the exchange interaction between adjacent grains, and thus reducing the media noise in the materials. The particles must also possess high *H*_C_ values to obtain high storage density [98]. The magnetic properties (*M*_R_, *M*_S_, and *H*_C_) of spinel ferrites depend upon the composition, particle size, crystal structure, and cationic distribution between octahedral and tetrahedral sites. Moreover, they may exhibit antiferromagnetic, ferrimagnetic and paramagnetic behavior [101,114].

The increase of *H*_C_ values may also occur due to a combination of spin disorder, spin canting effect, and improved surface barrier potential in surface layers [87,115]. In the case of magnetic NPs, the presence of a large number of atoms at the surface due to large surface-to-volume ratio results in interesting and superior properties in comparison to corresponding bulk materials. Moreover, the low number of coordination surface atoms relative to interior atoms of the nanoparticle leads various surface effects such as spin canting, spin disorder and existence of a magnetically dead layer [115].

High coercivity materials are known as hard materials, while low coercivity materials are known as soft materials [113]. The soft materials are used for inductor cores, transformers, and microwave devices, while hard materials are used for permanent magnets. Generally, the *H*_C_ value is low in soft ferrites and the magnetization can be tailored for cutting-edge applications in electronic engineering such as microwave components, high-frequency inductors and transformer cores [58]. Furthermore, due to the high *H*_C_ values of hard ferrites, it is not so easy to magnetize, and hence it is used as enduring magnets with applications in washing machine, refrigerator, communication systems, microwave absorbing systems, loudspeaker, TV, switch-mode power supplies, DC-DC converters, and high-frequency applications [58].

CoFe_2_O_4_ is a ferrimagnetic ceramic with excellent properties, such as high *K*, *H*_C_, and *T*_C_ values, moderate *M*_S_ values, and large magnetostrictive coefficient value [42,116]. The high *M*_S_ and *M*_R_ values can be explained on the basis of Néel’s theory and cation distribution at tetrahedral (A) and octahedral (B) sites [11,25,117]. Generally, in single-phase samples the magnetic characteristics are highly influenced by the material microstructure such as shape and size of crystals, residual stress and crystal defects [51]. Moreover, the synthesis temperature plays a key role in controlling particle size of CoFe_2_O_4_ with a significantly influence on its magnetic properties [66]. As expected, *M*_S_ values increase with the CoFe_2_O_4_ content and temperature, due to the increase of the crystallinity degree as well as to the average size of the magnetic CoFe_2_O_4_ NPs [14,42,99]. Due to the high values of *K* and high value of spin-orbit coupling constant of Co^2+^ ions, CoFe_2_O_4_ displays the highest magnetostriction (i.e., up to 200 ppm) among oxide-based materials. The increment of *H*_C_ value is mainly attributed to the increasing particle size as a consequence of the coarsening of nanocrystals and diminished crystal defects at high temperature [98]. In the case of CoFe_2_O_4_ embedded in SiO_2_ matrix (CoFe_2_O_4_@SiO_2_), the reduction of *M*_S_ and *M*_R_ values is attributed to the reduced amount of magnetic material per gram of nanocomposite [105]. Moreover, the increase of annealing temperature leads to the decrease of *K* and *H*_C_ values [74]. In contrast, the *H*_C_ value of CoFe_2_O_4_@SiO_2_ NPs does not show any change after coating, while the *H*_C_ value of MnFe_2_O_4_ NPs decreases by 10% after coating [105]. In the case of CoFe_2_O_4_ obtained by the citrate gel method, higher *M*_S_ values were obtained for samples synthesized at high temperatures than the samples prepared at low temperatures, suggesting that the *M*_S_ value rises with increase in particle size, while the *M*_R_ decrease with the increase of particle size [62]. Peixoto et al. [43] produced CoFe_2_O_4_ NPs embedded in a SiO_2_ matrix via sol–gel method and evaluated the magnetic properties at 5 K and 100–200 K, revealing a superparamagnetic behavior. 

The *M*_S_ value of 27.09 emu/g found for CuFe_2_O_4_ obtained by solid state chemistry (particle size of 36 nm) was similar to the *M*_S_ value of 33 emu/g for CuFe_2_O_4_ NPs of ~32 nm obtained by other synthesis method. However, the *H*_C_ of 112 Oe found for CuFe_2_O_4_ obtained by solid state chemistry (particle size 36 nm) is greater compared to other syntheses, as a consequence of the presence of some impurities (CuO). Further, an *M*_R_ value of 4 emu/g was reported for CuFe_2_O_4_ of particle size ~8 nm synthesized by the sol-gel method [113]. The *H*_C_ of CoFe_2_O_4_ NPs show a maximum *H*_C_ value at a particle size of about 25 nm, while in the case of CuFe_2_O_4_ NPs obtained by sol-gel, co-precipitation, solid-state reaction, thermal decomposition, and solution auto-combustion methods, the *H*_C_ decreases linearly with the decreasing size, indicating that the particles are in single-domain area and the surface spins dominate the magnetization decreases with size reduction [57]. The *H*_C_ and *M*_S_ values of CuFe_2_O_4_ were higher in the case of the combustion method and lower in the case of the precipitation method [57].

The increase of *M*_R_ and *M*_S_ values of MnFe_2_O_4_ can be ascribed to the metal cations distribution at octahedral and tetrahedral sites of crystal lattice structure, meaning to the tendency of Mn^2+^ ions to be positioned at octahedral (B-site) [118]. MnFe_2_O_4_ NPs and MnFe_2_O_4_@SiO_2_ nanocomposite exhibit superparamagnetic behavior attributed to the ultra-small size of MnFe_2_O_4_ NPs and are widely used for drug delivery applications. The low *M*_S_ value of MnFe_2_O_4_@SiO_2_ nanocomposite is due to the presence of non-magnetic SiO_2_ matrix [3]. The *H*_C_ of MnFe_2_O_4_@SiO_2_ NPs slightly decreases compared to that of native magnetic NPs. No similar behavior was observed for CoFe_2_O_4_@SiO_2_ probably due to the larger contribution of the surface anisotropy to the total anisotropy of MnFe_2_O_4_ NPs [105]. The *M*_S_ value of 52.4 emu/g obtained in the MnFe_2_O_4_ (diameter of ~15.9 nm) is lower than values 67.0 emu/g for synthesized MnFe_2_O_4_ crystallites using a triethanolamine-assisted route under mild conditions (diameter of ~1 μm), but higher than the value of ~48.6 emu/g for the polymer-pyrolysis route MnFe_2_O_4_ NPs (diameters of ~9 nm) [101].

ZnFe_2_O_4_ displays antiferromagnetic behavior when the temperature is below the Néel temperature and diamagnetic, superparamagnetic, or ferrimagnetic behavior when particle sizes are at nanometer scale [119]. The superparamagnetic behavior is due to the increased disorder of magnetic moments orientation in the various sites when the ratio surface/volume increases. The *M*_S_ value of ZnFe_2_O_4_ strongly depends on various factors, such as the synthesis route and its conditions, type of the precursors, and annealing treatments [63]. The *M*_S_ value of 7.06 emu/g for ZnFe_2_O_4_ (diameter of ~17.9 nm) is lower than values of 54.6 emu/g for hydrothermal-synthesized ZnFe_2_O_4_ ultrafine particles (crystallite size of ~300 nm) [101]. However, the paramagnetic behavior of ZnFe_2_O_4_ was remarked upon in bulk [46] and NPs, due to the increase of an atypical Fe^3+^ cation distribution in the tetrahedral coordination sites upon nanosizing [47,101].

In the crystal structure of NiFe_2_O_4_, the Ni^2+^ occupies the octahedral site with Fe^3+^ cations distributed equally at tetrahedral and octahedral sites, while their antiparallel spins produce a net magnetic moment of 2*μ_B_* owing to Ni^2+^ ions at octahedral site [87]. Due to high crystallinity and uniform morphology, NiFe_2_O_4_ ferrites show high *M*_S_ and low *H*_C_ values [119]. The magnetic properties of NiFe_2_O_4_ NPs are attributed to the cumulative effect of various factors, such as super-exchange interaction, magneto crystalline anisotropy, canting effect, and dipolar interactions on the NP’s surface. Besides, the variation of *H_C_* value with particle size can be explained by the domain structure, critical size and anisotropy of the crystal [20]. The large NiFe_2_O_4_ is due to the scattering in direction of anisotropy field due to inhomogeneous broadening, at high temperature, it has the tendency to make magnetic moment isotropic causes the decrease of *H*_C_ value [21]. The *M*_S_ value of 31.9 emu/g found for NiFe_2_O_4_ (particle size of ~8.2 nm) is close to the values of 34.5 emu/g for NiFe_2_O_4_ NPs (crystallite size of ~68 nm) obtained by egg-white solution route, but lower than the theoretical *M*_S_ of 50 emu/g calculated using Neel’s sublattice theory and the reported value of 56 emu/g for the bulk sample [96]. The *M*_S_ value of 55.3 emu/g obtained in the CoFe_2_O_4_ (diameter of ~8.5 nm) is lower than values of 80 emu/g for bulk CoFe_2_O_4_ and ~65 emu/g for the CoFe_2_O_4_ NPs with crystallite size of ~40 nm synthetized by aerosol route, and higher than the values of 30 emu/g for hydrothermal-synthesized CoFe_2_O_4_ NPs (diameter of ~30 nm).

The synthesis of nanocrystalline spinel ferrite of type MFe_2_O_4_ (e.g., M = Mn, Co, Ni, Cu, Zn) has great relevance to modern technological applications in several industrial and biological fields, including magnetic recording media and magnetic fluids for the storage and/or retrieval of information, magnetic resonance imaging enhancement, and magnetically guided drug delivery [101]. Furthermore, the magnetic NPs showed promising results in the field of medicine and healthcare treatment owing to their biocompatibility, low toxicity, and ability to be handled by the application of a magnetic field [50].

Table 1 presents the main magnetic parameters (*M*_S_, *M*_R_, *H*_C_, *K*), particle diameter, and synthesis method of the studied ferrites. 

The magnetostrictive materials are solids in which the application of a magnetic field results in strong strain and deformation as a consequence of the strong coupling of magnetic moments with crystal lattice [138]. The large magnetostriction in a wide range of temperature is very useful when a mechanical coupling is required in multiferroic composite systems [139]. The electronic and optical properties of magnetostrictive solid materials are more sensitive to magnetic field comparing to solids with weak magnetostriction [138]. Magnetostrictive smart materials are generally used to design sensors, actuators, sonar transducers, motors, etc. In this regard, there is an increasing interest on metal oxide based magnetostrictive materials such as CoFe_2_O_4_ due to various practical advantages over alloy based magnetostrictive materials, i.e., the high magnetostriction and strain-field derivative with low saturation field of CoFe_2_O_4_ results in potential for sonar detector and force sensor applications and vibration components in high frequency ultrasonic transducer [140,141,142]. The magnetostriction value of CoFe_2_O_4_ prepared using micron-size powders without magnetic annealing decreases from 130 ppm to 200 ppm [142]. The magnetic annealing may increase the magnetostriction and strain derivative values due to the uniaxial anisotropic distribution of magnetic domains, while various magnetic or non-magnetic ions could decrease the magnetostriction and increase the strain derivative values [142]. The application of a magnetic field to magnetostrictive ferrimagnetic CoFe_2_O_4_ leads to strong strain and deformation of the crystal lattice and to change of spectrum-strain-magneto-optics, while high magnetoreflection is remarked on in the case of great magnetostriction [138].

Beside the excellent soft magnetic properties, Fe-based soft magnetic composites have unique properties such as high electric resistivity and low eddy loss, making them good electrical insulation coatings without any notable decrease in magnetic properties [143]. *H*_C_ value confirms the magnetic properties of hard magnetic or soft magnetic materials. CoFe_2_O_4_ obtained using co-precipitation method and low heat treatment have *M*_S_ of 21.74 emu/g, *M*_R_ of 2.37 emu/g, and *H*_C_ of 556.57 Oe [144]. The in-situ oxidation is an effective and novel method for manufacturing soft magnetic composites with low core loss for applications in medium and high frequency fields. The nano-ZnFe_2_O_4_ layer effectively improves the magnetic properties of soft magnetic composites [143]. Soft magnetic spinel ferrite properties probably occur due to the interactions within metal oxide and particular vacancy oxygen ions in their cubic spinel structure [144]. 

### 3.2. Photoluminescent Applications

Room temperature photoluminiscence is one of the important properties of mixed spinels as CoFe_2_O_4_, NiFe_2_O_4_ and ZnFe_2_O_4_ nanostructures. The photoluminescence spectrum offers information regarding the surface oxygen vacancies and defects, as well as the efficiency of the charge carrier trapping, immigration and transfer [17]. The broad visible band emission was ascribed to the charge transfer among Fe^3+^ at octahedral sites, M^2+^ (M = Co, Ni and Zn) at both tetrahedral and octahedral sites, and its surrounding O^2−^ ions [1]. The blue emission peak at 460 nm is attributed to the Fe^3+^ transition in the ferrite sites, while the main peak at 418 nm resulted from the free electrons trapped at the oxygen vacancies. The intensity of every peak diminished with the increasing Ni content, as a consequence of the increasing band gap which reduces the electron-hole recombination ratio [145]. The violet emissions resulted from the radiating defects associated to the interface traps within the grain boundaries. The decrease in the luminescence intensity of Co^3+^ from CoFe_2_O_4_ with the increase of doping fraction suggests a lower electron-hole recombination ratio [81]. Photoluminescence measurements showed a value around 2.13 eV for the energy gap of nanocrystalline ZnFe_2_O_4_ [63].

### 3.3. Catalytic Applications

Spinel ferrites have been extensively used as heterogeneous catalysts as they can be simply recovered from the reaction mixture by filtration or in the presence of an external magnetic field and recycled numerous times, making the process more economically and environmentally feasible [53]. Heterogeneous catalytic nanomaterials play a central role in the selective protection of functional groups in order to be economically valuable and environmentally friendly. The catalytic property of MFe_2_O_4_ (M = Cu, Ni, Co, Zn) spinel revealed that CoFe_2_O_4_ catalyst displayed the best performance using benzaldehyde with 63% conversion and 93% selectivity, while CuFe_2_O_4_ catalyst using H_2_O_2_ oxidant showed 57.3% conversion and 89.5% selectivity [53]. The particle size, surface area, morphology, and chemical composition considerable influence the catalytic activity of materials [134]. In addition, the catalytic property of nanocrystalline spinel ferrites depends on the distribution of cations among the tetrahedral (A) and octahedral (B) sites. As catalyst, CoFe_2_O_4_ displays the best performance compared to other ferrites [13]. The catalytic activity of CoFe_2_O_4_ was successfully reported for the synthesis of arylidene barbituric acid derivatives. The advantages of this method were short reaction time, high yields, simple and economic work-up procedure, high turnover frequency, chemoselectivity, environmental sustainability, and green conditions [38]. Large-size magnetic CoFe_2_O_4_ NPs were successfully used as catalyst for the oxidation of various alkenes in the presence of tert-butylhydroperoxide. The catalyst from the medium was easily separated using an external magnet and the catalyst was recycled several times without important loss of activity. CoFe_2_O_4_ exhibited high catalytic activity and selectivity in methanol decomposition to CO and H_2_ [38]. Moreover, non-nanosized CoFe_2_O_4_ displayed catalytic applications, such as the conversion of CO to CO_2_. In the case of the catalytic oxidation of benzyl alcohol to produce a mixture of benzaldehyde, benzoic acid, and benzyl benzoate, the CoFe_2_O_4_ NPs have been proven to be highly selective [13]. CoFe_2_O_4_ NPs can be also used as catalyst for Knoevenagel condensation reactions between aromatic aldehydes and ethylcyanoacetate, in mild conditions. The catalyst could be easily recovered from the reaction mixture using an external magnet and can be re-used four times, without a significant loss of activity [69].

NiFe_2_O_4_ is an effective catalyst in several industrial processes. Its catalytic activity related to O_2_ yield and evolution rate under ambient reaction conditions, is comparable to that of Ir, Ru or Co-based materials [119]. Due to its importance for both industry and domestic safety, the catalytic combustion of CH_4_ has attracted considerable interest, because of its higher energy conversion efficiency and low emissions of environmental pollutants. Therefore, the development of a high activity catalyst with sensitivity for CH_4_ detection is of great interest [19]. In this regard, the catalytic performance of NiFe_2_O_4_ for CH_4_ combustion, but also for alkylation and oxidation reactions has been reported [19]. NiFe_2_O_4_ was successfully used also as a catalyst in photocatalytic water oxidation using [Ru(bpy)_3_]^2+^ as a photosensitizer and S_2_O_8_^2−^ as a sacrificial oxidant [119].

CuFe_2_O_4_ has been used in industrial processes as a catalyst in both organic and inorganic reactions [146]. The main advantages of CuFe_2_O_4_ NPs are simple work-up and low-cost procedure, mild reaction conditions, reusable catalyst, high yield, short reaction times, no isomerization during the reaction [38]. The use of CuFe_2_O_4_ NPs in organic catalysis, such as the reaction of substituted aromatic aldehydes, ethyl acetoacetate, and ammonium acetate, at room temperature was also reported. In all cases, the nano-catalyst can be easily recovered and re-used [9,38]. CuFe_2_O_4_ NPs were also employed as reusable heterogeneous initiator in the synthesis of 1,4-dihydropyridines, of α-aminonitriles and conversion of CO to CO_2_ [38,146]. The successful use of CuFe_2_O_4_ NPs as catalyst for the ligand free N-arylation of N-heterocycles and for the cross-coupling of aryl halides with diphenyl diselenide to produce diaryl selenides was also reported [147,148]. The CuFe_2_O_4_ NPs also proved to be efficient catalysts for a simple, one pot, green, and efficient synthesis method for substituted benzoxazoles by redox condensation [149]. 

ZnFe_2_O_4_ spinel acts as a wide band gap semiconductor with application in hydrogen generation by light-activated water splitting and in photocatalytic removal of harmful organic molecules [85]. Non-nanosized ZnFe_2_O_4_ have been used as catalysts in the oxidative conversion of methane, oxidative coupling of methane, and in methanol decomposition to CO and H_2_O [38]. Additionally, the coupling between the catalyst and magnetic material is a new approach to enhance the catalytic performance of a catalyst [150].

### 3.4. Photocatalytic Applications

Photocatalysts are important materials that support the use of solar energy in oxidation and reduction processes, with applications in various areas, such as the removal of water and air contaminants, odor control, bacterial inactivation, water splitting to produce hydrogen, the inactivation of cancer cells, etc. [151]. Nowadays, the usage of photocatalysis is the preferred treatment methods for dyes removal, as alongside irradiation of light on a semiconductor, the produced electron-hole pairs are used also for the oxidation and reduction process [81]. The degradation of dyes occurs due to the formation of active radicals during the photocatalytic reaction [22,145].

Only few materials are capable of both photo-oxidation and photo-reduction, namely to complete the decomposition of harmful organic compounds and, concomitantly, to absorb visible light efficiently. The low crystallite size of ferrites leads to a large surface area and more reaction sites, increasing the photocatalytic activity [151]. The type of photocatalyst, crystallinity, size of NPs, accessibility of the active surface to pollutants, and diffusion resistance of organic pollutants are the most important characteristics that enhance the photocatalytic properties. Thus, small particle sizes with high crystallinity are of great interest due to their higher specific surface area and active sites that favor the photocatalytic activity [37]. Due to their spinel crystal structure and a band gap capable to absorb visible light, ferrites are suitable as photocatalysts for the degradation of a wide range of contaminants [151].

CoFe_2_O_4_ was used as photocatalyst for the degradation of organic dyes (i.e., methylene blue (MB) and rhodamine B (RhB)), its chemical stability and the narrow bandgap (1.1–2.3 eV) making it active under visible light [81,145]. CuFe_2_O_4_ photocatalyst was found to be more effective than ZnFe_2_O_4_ and NiFe_2_O_4_ in decomposing hazardous dye compounds, probably due to lower crystallite size, large surface area, small band gap and occurrence of pores that trap oxygen molecules and produce large number of oxidizing species and OH• radicals. The photocatalyst was recovered using a magnet and reused in four consecutive cycles under visible light [22]. CuFe_2_O_4_ “oversized” nanostructures displayed improved photocatalytic activity in the conversion of benzene under Xe lamp irradiation. Moreover, CuFe_2_O_4_ powders exhibited a good catalytic efficiency (~60%) at 200 °C and pH = 12 due to the high surface area (~120 m^2^/g) [38]. The photocatalytic ozonation of dyes with CuFe_2_O_4_ NPs prepared by co-precipitation method was reported to successfully decolorize and degrade textile dyes (Reactive Red 198 and 120) without using high pressure of oxygen or heating [152].

The photocatalytic activity of ZnFe_2_O_4_ NPs relies on the surface properties and defects. ZnFe_2_O_4_ was tested for solar energy conversion and photochemical hydrogen production due to its relatively narrow band gap energy (1.9 eV), excellent visible-light response, good photochemical stability and low-cost [17]. The use of ZnFe_2_O_4_ NPs for the photocatalytic degradation of 4-chlorophenol and the dependence of the degradation efficiency on their particle size and morphology was also reported [134]. ZnFe_2_O_4_ was found to be an appropriate visible-light-driven catalyst, which completely degraded the RhB dye in short time, due to the small particle size and narrow size distribution [107]. The sol–gel/precipitation hybrid synthesis method of magnetic NiFe_2_O_4_@TiO_2_ and its use as photocatalyst for the production of hydrogen from aqueous systems was reported by Kim et al. [153].

### 3.5. Water Decontamination

In the last years, the industrial wastewater management is one of the main challenges in developed countries. Wastewaters resulted from the textile industry contain many non-biodegradable organic dyes mixed with different contaminants in a variety of ranges. Untreated effluents negatively affect not only humans and animals, but also the flora and fauna [81]. RhB is a synthetic, highly toxic, water soluble, organic dye, widely used as colorant in many industries and frequently found in wastewaters. Various techniques, such as ozonation, electrochemical method, and Fenton process, have been employed for the treatment of RhB containing water. The use of CoFe_2_O_4_ for wastewater treatment is based on its high adsorption capacity, magnetic properties that allow the NPs separation using an external magnetic field, and on its low energy band gap that enables the photocatalytic degradation of RhB under visible light irradiation, [37,81]. Moreover, CuFe_2_O_4_ is considered a prospective material for the water decontamination by photoelectrochemical processes. In combination with a suitable adsorbent, CuFe_2_O_4_ can be applied as a cost-effective adsorbent for removal of MB from water and wastewater [146]. The atrazine degradation by a three-dimensional electrochemical process using CuFe_2_O_4_ NPs as electrodes and catalyst for the activation of persulfate for were also reported [149]. Further, ZnFe_2_O_4_ can be effectively used as magnetically recyclable material for the removal of chemical contaminants (i.e., dyes), as well as biological contaminants from water/industrial wastewater treatment [154].

### 3.6. Coloring

Ceramic pigments are metal transition oxides with high thermal and chemical stability, high tinting strength when dispersed and fired with glazes or ceramic matrices, high refractive index, acid and alkali resistance, and low abrasive strength. The color of each pigment is obtained by adding chromophore agents (usually transition metals) in an inert oxide matrix [155]. 

CoFe_2_O_4_ is a black pigment widely used in the ceramic industry. The color performance depends on the coating crystallization degree, a larger number of crystals in the glass resulting in a brighter color [156]. The ZnFe_2_O_4_ spinels are thermally stable, insoluble, and resistant to aggressive media, exhibit a high covering power and a reasonable cost. ZnFe_2_O_4_ spinel pigments improve the mechanical strength of the binder by a chemical reaction producing cationic soaps, resulting in low solubility and a tendency towards saponification in contact with a corrosive environment [157]. The color of ZnFe_2_O_4_ depends on the annealing temperatures and particle size as it causes a reduction of the total reflecting surface of the powder. At high annealing temperatures, the system suffers a notable modification due to the disappearance of defects (as oxygen vacancies), leading to less distorted tetrahedral and octahedral sites, and consequently better-defined colors [158]. The possibility to select the desired cations in the spinel lattice (Zn^2+^ in A sites and Fe^3+^ in B sites for ZnFe_2_O_4_) can be used to change or to create new properties of the pigment dispersed in an organic binder [157]. 

### 3.7. Corrosion Protection

Metal corrosion is caused by an electrochemical process, generally promoted by Cl^−^ ions, which dissolve Fe oxides [159]. The corrosion of a metallic substrate may be constrained by the transformation of ferrous (Fe^2+^) ions to ferric (Fe^3+^) ions, which act as inorganic corrosion inhibitors in the electrochemical process, leading to the formation of a passivation layer_,_ which will overcome the cathodic and/or anodic reaction [157]. 

Organic coatings act as barrier or mechanical protection against corrosive environments [157]. Nanocontainers can release corrosion inhibitors and subsequently protect the metal substrate from corrosion when incorporated into an organic coating [160]. The CoFe_2_O_4_@SiO_2_ nanopigment enhances the corrosion protection performance of the coating better than CoFe_2_O_4_, by its appropriate dispersion in the coating and by filing and blocking the free cavities and all electrolyte pathways in the coating. Besides, it releases inhibitive species (Co^2+^ cations) in the scratched area and restricts corrosion inhibition at the coating/metal interface. The incorporation of CoFe_2_O_4_@SiO_2_ nanopigment into epoxy coatings results in a noticeable improvement of the coating corrosion protection [160]. Furthermore, the corrosion protection of pigments, such as diatomite, talc, wollastonite, and kaolin, was considerably improved using the surface treatment with a layer of ZnFe_2_O_4_ on the particle surface. Low amounts (16–20 wt.%) of ZnFe_2_O_4_ used for the surface treatment also enhance the corrosion protection [157]. 

### 3.8. Sensors

Sensors are devices used for signaling changes that appear in a specific material in a certain environment [40]. Sensors based on ferrite NPs are highly sensitive, have low detection limits, and a high signal to noise ratio [40]. One of the most frequent uses of sensors is for detecting changes of humidity. The monitoring of humidity is frequently used in industrial and domestic environments in order to assure human comfort and the appropriate storage of various goods, as well as the optimal functioning condition for industrial process and various instruments [161]. Generally, the humidity-sensing phenomenon is based on the surface effect of water vapor–solid interaction. The ceramic type humidity sensors based on metal oxides displayed superior advantages comparing to polymer films in terms of physical stability mechanical strength, thermal capability, and resistance to chemical attack, making them promising materials for electrochemical humidity sensor applications. By water adsorption, the electrical properties (i.e., capacitance, resistance or electrolytic conduction) of the ceramic surfaces change. In this regard, by increasing the humidity, the conductivity increases, resulting in a higher dielectric constant [145]. A ceramic thick film humidity sensor of MnZn ferrite based on interdigitated electrodes has been reported by Arshaka et al. [162].

The humidity sensing efficiency of a material depends on its microstructural characteristics that are related to the synthesis method [161]. In this sense, the higher sensitivity of ZnFe_2_O_4_ can be attributed to the small grain size, high surface area accessible for adsorption of water vapor and is correlated with the low barrier height. Nanosized CoFe_2_O_4_, CuFe_2_O_4_ and NiFe_2_O_4_ have also been demonstrated to be good sensors for detecting oxidizing gases such as chlorine [60]. Owing to its high *H*_C_ values and stability, the sensors and actuators made by CoFe_2_O_4_ are more durable and of extensive use [7]. Magnetostrictive CoFe_2_O_4_ composites attracted interest for the development of magnetoelastic sensors based on high magnetostriction, response to applied stress, chemical inertness and low cost [163]. Furthermore, due to the bifunctional nature in terms of stress sensing and actuation or contraction under the influence of a magnetic field, CoFe_2_O_4_ has been classified as a smart material with various technological applications among position sensors [120].

Fiber optic biosensors offer great advantages over conventional sensors. Due to its technological advantages (high stability, possibility to be easily removed from the reaction medium, and reuse), the NPs immobilized in glucose oxidase are of great significance for the development of fiber optic glucose oxidase sensors, widely used in food technology, fermentation products, and glucose biosensor [164]. Besides, the immobilization of glucose oxidase sensor on functionalized CoFe_2_O_4_@SiO_2_ NPs via cross-linking with glutaraldehyde can be an effective way to produce the ideal immobilized enzyme [164].

A carbon paste electrode containing ZnFe_2_O_4_ NPs was proven to have high sensitivity and good reproducibility in detecting trace levels of 5-fluorourcile in drug samples [165]. 

### 3.9. Dielectric Applications

Generally, the dielectric structure involves well conducting grains, separated by low conductivity grain boundaries [166,167]. The dielectric properties of spinel ferrites are determined by structural homogeneity, cation distribution, particle size, density, and porosity [167]. These properties are highly dependent on the synthesis method and thermal treatment parameters, such as temperature, duration, or heating and cooling rate [74]. 

In case of CoFe_2_O_4_, the small Co^2+^ ion occupying the tetrahedral site, reduces the lattice constant, facilitates the electron hopping between Fe^3+^ and Fe^2+^ ions and acts as a source of charge carrier enhancing the dielectric behavior of CoFe_2_O_4_ [8]. At low frequencies, the polarization in CoFe_2_O_4_ is determined by the hopping of charge carriers, that accumulates and produces polarization upon reaching the grain boundaries, leading to high dielectric constants. In contrast, at the higher frequencies range, the hopping of charge carriers is not able to follow the alternating current induced field, and are thus incompletely polarized, resulting low dielectric constants [166,167]. The dielectric constant decreases by increasing the grain size, which further decreases the grain boundary between the small grains [118]. The dielectric constant is high at lower frequencies, and decreases with increase of frequency [167]. This decrease becomes constant beyond a certain frequency as only the dielectric polarization contributes to dielectric constant [25]. The dielectric constant gradually also increases with increasing temperature due to dielectric polarization [168]. At low temperature, the charge carriers are unable to orient along the applied electric field direction, therefore the polarization is weak and the dielectric constant is very low [168]. However, as temperature increases, a high number of charge carriers liberate and contribute to polarization, that further increases the dielectric constant [168]. The charge carriers exchange between different valence states of elements present in nanosized ferrites highly influences the polarization [166]. High dielectric constants at high frequencies are attributed to the occurrence of space charge polarization due to inhomogeneous dielectric structure of grain size and impurities [169]. Gopalan et al. [170] reported lower dielectric constant values of CoFe_2_O_4_ NPs prepared by the sol-gel method compared to bulk CoFe_2_O_4_. The CoFe_2_O_4_@SiO_2_ NCs with controlled magnetic and dielectric properties are promising candidates for biological and high frequency applications [30]. CuFe_2_O_4_ is a potential candidate for microwave devices due to the adequate magnetic and dielectric properties at high frequency [27]. 

NiFe_2_O_4_ has also a dielectric structure with grains and grain boundaries of different conducting properties [171]. The exchange electron between Fe^2+^ and Fe^3+^ ions and the hole that transfer between Ni^3+^ and Ni^2+^ ions assure the electrical conduction and dielectric polarization [171]. By increasing the frequencies, the electron/hole exchange frequency will not be able to follow the applied electric field, resulting in lower polarization [171]. 

### 3.10. Antimicrobial Applications

Iron plays a key role in microbial pathogenesis, many organisms using Fe sequestration as a defense against infection [172]. The Fe(II)- and Fe(III)-resistant microorganisms showed unexpected resistance to a range of antibiotics such as ampicillin, chloramphenicol, rifampicin, sulfanilamide and tetracycline. Ferrite NPs exhibit good antibacterial properties against *Bacillus* *cereus, Escherichia coli, Staphylococcus aureus, Pseudomonas aeruginosa*, *Serratia marcescens,* and *Candida albicans*. The strain of *Escherichia coli* was capable of evolving resistance to excess Fe mainly through changes in the uptake of Fe [172].

CoFe_2_O_4_ NPs are well-known candidates for biomedical applications due to the antimicrobial activity against many human pathogens [55]. The high surface-to-volume ratios and nanoscale size of CoFe_2_O_4_ NPs improves their reaction with the pathogenic microbes [173]. CoFe_2_O_4_@SiO_2_@Ag nanocomposite and its combination with Streptomycin exhibited significant antibacterial activity against both Gram-positive and Gram-negative bacteria. The deposition of Ag NPs on CoFe_2_O_4_@SiO_2_ can prevent agglomeration. Moreover, it can be easily recovered from solution after disinfection, by applying of an external magnetic field due to the presence of magnetic core in the composite [174].

CoFe_2_O_4_ adheres to the membranes of microorganisms, thus increasing the lag stage of the bacterial growth period, spreading the production time of microorganisms and increasing the bacterial cell division. The antimicrobial activity of CoFe_2_O_4_ is due to its produced effective oxides (i.e., superoxide (O^2−^) and hydrogen peroxide (H_2_O_2_)) [173]. The ferrite NPs antibacterial effect on unicellular fungi is dependent on the diffusion flow of an effective oxide within the bacteria cell surface [173].

The antimicrobial activity of CoFe_2_O_4_ NPs was studied against multidrug resistant clinical pathogens (*Staphylococcus aureus, Escherichia coli*, *Candida parapsilosis* and *Candida albicans*) by assessing the colony forming units [175,176]. Furthermore, CoFe_2_O_4_ NPs functionalized with oleine (CoFe_2_O_4_@Ole) and lysine (CoFe_2_O_4_@Lys) demonstrated high efficiency against all tested microorganisms most probably due to (i) CoFe_2_O_4_@Lys NPs can easily interact electrostatically with negatively charged *S. aureus* and *E. coli* cell walls, causing destruction of the cytoplasm; (ii) positively charged CoFe_2_O_4_@Lys NPs and negatively charged CoFe_2_O_4_@Ole NPs by can affect bacteria cell homoeostasis. The high antimicrobial efficiency of CoFe_2_O_4_@Ole NPs could be due to their higher stability compared to CoFe_2_O_4_@Lys NPs or to a negative curvature wrapping of anionic membranes [175].

The ZnFe_2_O_4_ nanomaterials also display good antibacterial activity against *Lactobacillus*, *Bacillus cereus* (Gram-positive), *Escherichia coli*, *Aeromonas hydrophila* and *Vibrio harveyi* (Gram-negative) bacterial strains [145,146,155]. The low crystallite size and surface area play a critical role in the antimicrobial activity against tested pathogenic microbes. The ZnFe_2_O_4_ NPs had no activity against *Escherichia coli* and was only active against *Staphylococcus aureus* and *Pseudomonas aeruginosa* [173]. 

NiFe_2_O_4_ had no activity against *E. coli*, but was active against *S. aureus* and *P. aeruginosa* [176]. In addition, the antibacterial effect of NiFe_2_O_4_@carbon nanocomposite on the degradation of *P. aeruginosa* bacteria was rapid and sensitive [95].

### 3.11. Biomedical Applications

The biomedical applications require non-agglomerated and stable aqueous dispersion of magnetic NPs with high *M*_S_ values and good biocompatibility [177]. The magnetic NPs can be highly effective for magnetic fluid hyperthermia, drug release and thermal excitation of metabolic pathways within a single cell [178]. In this regard, the MnFe_2_O_4_ NPs have attracted considerable attention in biomedicine due their easy synthesis process, controllable size, high magnetization value, superparamagnetic nature, ability to be monitored by external magnetic field, surface manipulation capability, and greater biocompatibility [3,179]. Moreover, the surface modification of MnFe_2_O_4_ NPs with mesoporous SiO_2_ or its incorporation into mesoporous SiO_2_ nanosphere could play an important role in the stabilization of NPs in water, in the enhancement of its biocompatibility, and in reducing the agglomeration and degradation of MnFe_2_O_4_, respectively [3]. 

The physical characteristics of CoFe_2_O_4_, especially high stability, led to extensive attention regarding potential biomedical applications [173]. Generally, CoFe_2_O_4_ has been used for drug delivery, imaging factor and therapy of brain tumors [173]. At high concentrations of CoFe_2_O_4_ NPs, metal ions are released and some of them enter the cells leading to cytotoxicity, according to the Trojan horse mechanism. A possible explanation could be the prevention of cell transcription and protein synthesis and subsequent altered cellular function [31]. The biocompatible SiO_2_ coating could remove or reduce the adverse effects. By coating CoFe_2_O_4_ NPs, their adhesion decreased due to the negative charge of SiO_2_ matrix, and they displayed superparamagnetic behavior with a low *M*_S_ value and higher compatibility, which made them suitable for various medical applications (drug delivery, specifically cancer cells, magnetic resonance imaging contrast agent for cancer diagnosis) [31,180].

Hyperthermia is a noninvasive treatment procedure for oncological pathologies in which both healthy and carcinoma cells of a living tissue are exposed to necrosis, by prolonged overheating (>43 °C) [181]. The existing tumor heating methods (hot water, microwave, infrared ray, ultraviolet ray, etc.) are ineffective for deep-seated cancers, but the magnetic induction hyperthermia, i.e., via magnetic NPs, may be effective in these types of tumor due to the selective heating and destruction of tumor tissue, with minimum collateral damage [178,182]. Magnetic hyperthermia is an emerging adjuvant therapy for malign tumors, where the appropriate magnetic NPs are located under a magnetic field and the heat released may remove the cancerous tissue, at an optimum temperature of 41–46 °C [15,123]. Ferrimagnetic materials own hysteretic properties under time-varying magnetic field, which lead to magnetically induced heating [183]. Moreover, the magnetic NPs have low toxicity, are biocompatible and well-tolerated by the living organisms, leading to a simple and fast analysis of specific absorption rate [177]. Some studies reported an effective technique of inserting ferrimagnetic NPs in a tumor region based on the magnetophoresis, a phenomenon caused by a magnetic field gradient on magnetically induced magnetic moment of particle [180,183]. Moreover, magnetophoresis is used in numerous commercial and industrial processes for the separation of magnetic NPs dangling in fluids [183]. 

However, the main challenge of hyperthermia is to lower the damage to nearby normal tissues. In this regard, NPs must be concentrated at the tumor site rather than in the tumor surroundings [50]. The uncoated magnetic NiFe_2_O_4_ NPs have the potential to be used in cell differentiable hyperthermia agents due to their high value of cell survival rate (85%) and non-cytotoxicity under different pH levels (pH = 7 normal cell and pH = 6 tumor cell) [15]. Further, ZnFe_2_O_4_ is a good candidate for hyperthermia due to its low toxicity [15,123]. The nanosized ferrite spinel’s enzyme-like activities include peroxidase, oxidase, and catalase, and their applications as enzyme mimetics in biosensing, molecular detection, cancer therapy, and drug delivery were recently reviewed [184]. 

The hyperthermia measurements CoFe_2_O_4_ and MnFe_2_O_4_ NPs were performed under various conditions, in order to normalize the obtained results by removing their dependence on magnetic field frequency and amplitude. MnFe_2_O_4_ NPs obtained in gelatin medium at 175 °C display the best reported heating efficiency, with values comparable to those of commercial magnetic NPs [185]. CoFe_2_O_4_ NPs with narrow size distribution and small particle size (~10 nm), and hence small hydrodynamic diameter, are successfully used to produce high magnetic hyperthermia within short duration [181]. Moreover, the magnetic CoFe_2_O_4_@MnFe_2_O_4_ NPs have proven to be superior to the individual NPs for producing effective heating in hyperthermia [178].

ZnFe_2_O_4_ type bioactive glass ceramics possess both the magnetic properties that generate adequate heat and the ability to bond to natural tissues (via hydroxyapatite layer) [182]. Consequently, this type of materials can be used for the hyperthermia treatment of cancer, but also as a substitute for a cancerous/ damaged bone. Such magnetic heat generation materials depend on various factors such as the structure and magnetic characteristics of the material, strength and frequency of alternating magnetic field, quantity of implant, etc. [182].

The synthesis of CoFe_2_O_4_@DMSA/DOX NPs to improve the efficiency of magnetic CoFe_2_O_4_ in therapeutic applications, by functionalizing its surface with meso-2,3-dimercaptosuccinic acid, followed by conjugation with the anticancer agent, doxorubucin was also reported [186]. As expected, the experimental results demonstrate that the combined thermal and chemotherapy effect induced by CoFe_2_O_4_@DMSA/DOX NPs displays an excellent antitumor efficacy by mitochondrial membrane disruption [186]. The well dispersed spherical magnetic ZnFe_2_O_4_ NPs exhibit a better heat efficiency compared to the aggregated polyhedral magnetic ZnFe_2_O_4_ NPs, being able to produce a threshold hyperthermia temperature of 42–45 °C in a short time, a key feature for magnetic hyperthermia applications [187].

The cytotoxicity of NPs considered as potential drug delivery systems must be taken into account, especially since it may vary with the type of cell line, exposure dosage, exposure time, particle size and aggregation tendency [188]. The in vitro estimation of cytotoxicity using different cancer cell lines (HeLa, cervical cancer cell and PC-3, prostate cancer cell) revealed good biocompatibility of NiFe_2_O_4_ NPs synthesized by co-precipitation and subsequent thermal annealing and less toxic effect to normal cell L929 [188].

The superparamagnetic response and biocompatibility of magnetic NPs make them potential candidates as contrast agents in magnetic resonance imaging (MRI) and tracer agents in magnetic particle imaging (MPI). Due to its soft magnetic behavior, NiFe_2_O_4_ is used as a contrast agent in MRI [189]. NiFe_2_O_4_@PAA composites (PAA - polyacrylic acid) could be considered as potential tracer agents for MPI, outperforming the commercial, commonly used tracer agents due to the minimum relaxation time of 3.10 μs and high resolution of 7.75 mT, [189]. Besides its use in photothermal and sonodynamic therapy of melanoma, MnFe_2_O_4_/C nanocomposite may also be used as a novel theranostic agent in nanomedicine [190]. In this regard, the intratumoral injection of MnFe_2_O_4_/C may induce deep tumor tissue necrosis, while the imaging studies endorse MnFe_2_O_4_/C as a contrast agent for MRI with a dose-dependent decrease in the signal intensity [189,190]. However, comprehensive studies on the in vitro and in vivo biodegradability and pharmacokinetics of MnFe_2_O_4_/C nanocomposite are necessary for additional investigations and evaluation [189,190].

## 4. Conclusions

Nanosized CoFe_2_O_4_, MnFe_2_O_4_, ZnFe_2_O_4_, NiFe_2_O_4_, and CuFe_2_O_4_ NPs have received impressive attention in the last decade due to their special properties, such as chemical, mechanical, and thermal stability, large coercivity, high anisotropy constant and Curie temperature, moderate saturation magnetization, high electrical resistance, and low eddy current loss. Amongst all the reviewed synthesis methods, the sol-gel and chemical co-precipitation techniques stand as superlative routes for synthesizing fine, homogenous, nanostructured ferrites. However, several unconventional methods allow the cost-efficient preparation of high-quality NPs. Although bulk ferrites remain a key group of magnetic materials, due to their advantageous properties, the investigated nanostructured ferrites are used in many areas, including material sciences, engineering, physics, chemistry, biology, and medicine. Notably, these magnetic ferrite NPs have displayed outstanding results in the field of medicine because of their low toxicity, biocompatibility, and ability to be easily manipulated using a magnetic field. 

## Figures and Tables

**Table 1:** Average crystallites size (*D*), saturation magnetization (*M*_S_), remanent magnetisation (*M*_R_), coercivity (*H*_C_) and anisotropy constant (*K*) of CoFe_2_O_4_, CuFe_2_O_4_, MnFe_2_O_4,_ NiFe_2_O_4_, ZnFe_2_O_4_, produced by different synthesis methods; RT-room temperature.

Ferrite	Synthesis Method	Temp.	*D_XRD_* (nm)	*M*_S_ (emu/g)	*M*_R_ (emu/g)	*H*_C_ (kOe)	*K*∙10^3^ (erg/cm^3^)	Ref.
CoFe_2_O_4_	sol-gel	RT	28.0	26.3	11.9	1.440	2.378	[46]
	sol-gel auto-combustion	RT	36.0	89.0	20.0	0.650	-	[11]
	sol-gel auto-combustion	RT	52.0	65.6	71.9	1.117	-	[78]
	solid-state	RT	80.0	77.2	-	0.525	-	[120]
	co-precipitation	RT	17.3	78.6	15.8	0.778	-	[121]
	co-precipitation	RT	37.0	72.6	34.5	1.060	-	[117]
	co-precipitation	RT	11.70	58.4	12.45	0.286	-	[108]
	combustion	RT	52.0	52.6	20.8	1.274	-	[122]
	hydrothermal	RT	17.3	63.4	23.1	0.831	0.450	[123]
	hydrothermal	RT	8.5	55.3	-	0.074	-	[101]
	hydrothermal	RT	16.3	58.3	-	1.029	-	[121]
	normal micelles	RT	5.58	12.6	0.17	0.0237	-	[108]
	reverse micelles	RT	7.62	29.4	0.84	0.0252	-	[108]
	reverse micelle	RT	6.00	34.4	8.50	0.500	-	[124]
	polyol	RT	14.0	57.0	21.6	1.123	-	[125]
	thermal decomposition	RT	13.0	68.0	-	0.560	-	[88]
	thermal decomposition	RT	82.8	48.38	11.72	0.64	-	[126]
CuFe_2_O_4_	sol-gel	RT	60.0	14.5	3.03	0.018	0.163	[46]
	sol-gel	RT	8.00	16.5	4.00	0.450	-	[113]
	sol-gel auto-combustion	RT	18.6	78.9	35.9	0.705	0.569	[127]
	solid-state	RT	38.0	27.1	1.41	0.112	-	[113]
	co-precipitation	RT	31.0	22.9	1.12	0.114	-	[113]
	co-precipitation	RT	52.0	28.1	8.52	0.189	-	[117]
	combustion	RT	22.3	14.0	0.17	0.006	-	[118]
	auto-combustion	RT	22.0	18.9	1.51	0.140	-	[113]
	thermal decomposition	RT	53.0	29.0	0.91	0.102	-	[113]
	thermal decomposition	RT	10–250	0.90	-	0.600	-	[128]
	sonochemical	RT	40.0	21.5	12.6	0.235	-	[24]
MnFe_2_O_4_	sol-gel	RT	49.0	28.5	14.8	0.119	3.392	[46]
	co-precipitation	RT	6.10	13.5	1.24	0.046	-	[129]
	hydrothermal	RT	22.0	65.5	3.86	0.054	0.018	[130]
	hydrothermal	RT	15.9	52.4	-	43.9	-	[101]
	hydrothermal	RT	25.4	67.3	-	0.173	-	[124]
	polyol	RT	7.10	51.9	-	-	-	[130]
	sonochemical	RT	25.5	59.4	3.41	0.024	1.53	[131]
	microwave combustion	RT	27.9	61.0	11.4	0.064	-	[53]
	thermal decomposition	RT	61.0	56.0	10.0	0.08	-	[132]
NiFe_2_O_4_	sol-gel	RT	23.0	20.1	6.82	0.061	0.770	[46]
	sol-gel	RT	70.0	37.3	-	0.321	-	[133]
	sol-gel auto-combustion	RT	58.0	50.0	7.00	0.050	-	[11]
	co-precipitation	RT	17.3	43.9	16.6	0.051	-	[121]
	combustion	RT	25.0	30.2	4.00	0.159	-	[122]
	microwave combustion	RT	18.5	37.9	2.57	0.016	-	[134]
	hydrothermal	RT	8.20	31.9	-	0.007	-	[101]
	thermal decomposition	RT	25.0	36.5	10.6	0.263	-	[87]
	thermal decomposition	RT	79.0	43.60	17.63	0.645	-	[135]
	thermal decomposition	RT	10.7	36.8	-	-	-	[136]
ZnFe_2_O_4_	sol-gel	RT	49.0	10.8	1.67	0.015	0.102	[46]
	sol-gel auto-combustion	RT	54.0	5.31	0.08	0.113	-	[78]
	solid-state	RT	80.0	77.27	-	0.525	-	[120]
	co-precipitation	RT	4.80	11.9	0.01	0.004	-	[129]
	microwave combustion	RT	37.5	2.60	0.01	0.007	-	[53]
	microwave combustion	RT	21.1	3.85	0.51	0.010	-	[134]
	hydrothermal	RT	15.9	52.4	-	0.044	-	[101]
	solvothermal	RT	80.0	77.0	-	0.090	-	[102]
	reverse micelle	RT	8.30	4.90	0.01	0.010	-	[107]
	thermal decomposition	RT	30.0	12.8	-	-	-	[137]

## Data Availability

The data presented in this study are available on request from the corresponding author.
